# The DQB1^*^03:02 Genotype and Treatment for Pain in People With and Without Multiple Sclerosis

**DOI:** 10.3389/fneur.2020.00993

**Published:** 2020-09-04

**Authors:** Sarah Burkill, Kelsi A. Smith, Pernilla Stridh, Ingrid Kockum, Jan Hillert, Hannes Lindahl, Lars Alfredsson, Tomas Olsson, Fredrik Piehl, Scott Montgomery, Shahram Bahmanyar

**Affiliations:** ^1^Department of Medicine Solna, Centre for Pharmacoepidemiology, Karolinska Institutet, Solna, Sweden; ^2^Clinical Epidemiology Division, Department of Medicine Solna, Karolinska Institutet, Solna, Sweden; ^3^Saw Swee Hock School of Public Health, National University of Singapore, Singapore, Singapore; ^4^Institute of Environmental Medicine, Karolinska Institutet, Solna, Sweden; ^5^Department of Clinical Neuroscience, Karolinska Institutet, Solna, Sweden; ^6^Centre for Molecular Medicine, Karolinska University Hospital Solna, Solna, Sweden; ^7^Centre for Occupational and Environmental Medicine, Stockholm County Council, Stockholm, Sweden; ^8^Clinical Epidemiology and Biostatistics, School of Medical Sciences, Örebro University, Örebro, Sweden; ^9^Department of Epidemiology and Public Health, University College London, London, United Kingdom

**Keywords:** epidemiology, genetics, cohort study, neurology, multiple sclerois

## Abstract

Murine models have demonstrated that the major histocompatibility complex (MHC) is associated with pain-like behavior in peripheral nerve injury, however, the same association has not been shown when considering injury to the central nervous system (CNS), which more closely mimics the damage to the CNS experienced by MS patients. Previous research has indicated the DQB1^*^03:02 allele of the class II HLA genes as being associated with development of neuropathic pain in persons undergoing inguinal hernia surgery or with lumbar spinal disk herniation. Whether this HLA allele plays a part in susceptibility to pain, has not, as far as we are aware, been previously investigated. This study utilizes information on DQB1^*^03:02 alleles as part of the EIMS, GEMS, and IMSE studies in Sweden. It also uses register data for 3,877 MS patients, and 4,548 matched comparators without MS, to assess whether the DQB1^*^03:02 allele is associated with prescribed pain medication use, and whether associations with this genotype differ depending on MS status. Our results showed no association between the DQB1^*^03:02 genotype and pain medication in MS patients, with an adjusted odds ratio (OR) of 1.02 (95% CI 0.85–1.24). In contrast, there was a statistically significant association of low magnitude in individuals without MS [adjusted OR 1.18 (95% CI 1.03–1.35)], which provides support for HLA influence on susceptibility to pain in the general population. Additionally, the effect of zygosity was evident for the non-MS cohort, but not among MS patients, suggesting the DQB1^*^03:02 allele effect is modified by the presence of MS.

## Introduction

Multiple sclerosis (MS) is an immune mediated, inflammatory disease, with selective targeting of the central nervous system (CNS). Individuals with MS experience a wide variety of symptoms, one of which is pain. Recently, we reported that MS patients are affected by pain, in particular neuropathic pain, to a greater extent than the general population through considering prescribed pain medications as a proxy for a pain diagnosis (1). The neuropathic pain in MS likely stems from the disease inherent lesions in the CNS (2). It is not clear why some patients develop neuropathic pain and some do not. It may be a stochastic phenomenon depending on where in the nervous system a person has inflammatory lesions. Additionally, the observed increased risk of pain in MS patients could be attributed to genetic susceptibility for this MS sub-phenotype. In MS *per se*, there is well-documented evidence for a genetic association with MS involving the major histocompatibility complex (MHC), in particular the human leukocyte antigen (HLA) with the strongest risks associating to carriage of the disease-predisposing HLA-DRB1^*^15:01 class II allele and to non-carriage of the protective HLA-A^*^02:01 class I allele (3). Moreover, numerous non-HLA genes of which the vast majority connect to inflammatory pathways, are associated with low odds ratios for MS (4, 5). The DQB1^*^03:02 gene has itself been shown to be associated with MS development (3). However, any genetic influence on pain sub-phenotypes, and if these differ from the ones associated with MS itself has not been reported so far. It is valid to study this in MS in view of experimental data on non-specific damage to the CNS and peripheral nervous system, indeed showing both MHC and non-MHC genetic influences on the development of pain (6–8). In humans, regarding damage to the peripheral nervous system, the DQB1^*^03:02 allele of HLA class II has been associated with development of neuropathic pain in persons that have undergone inguinal hernia surgery or with lumbar spinal disk herniation (9). The DQB1 gene codes for the beta chain of the cell-surface protein receptor, HLA-DQ, which is a heterodimer on cells presenting antigen to CD4+ T cells, and allelic differences determine the spectrum of peptides presented (10). Pain is an important sub-phenotype in MS since it can lead to decreases in physical abilities and reduction in quality of life (2). There are several treatments for pain prescribed to MS patients, such as tramadol and some opioids (11) which are also often prescribed to the general population without MS. There are also drugs used specifically for neuropathic pain, including pregabalin and gabapentin, which our previous study used to identify instances of neuropathic pain (1).

In view of previous human data on the HLA association with peripheral neuropathic pain, in this study we focused on the question of whether carriers of the allele DQB1^*^03:02 had an increased risk of pain and if such an effect would be different in MS patients and those without MS. Both general pain and neuropathic pain can be identified using prescriptions of pain medication as a proxy, which can identify pain with a reasonable degree of accuracy, (1) and we examined these categories of pain using the same definitions in those with and without MS. Zygosity of DQB1^*^03:02 could also give insight into whether there is a dose-dependent association with the likelihood of experiencing pain, and whether this association differs for those with and without MS. We also assessed whether this association is the same for neuropathic pain, which is particularly common among MS patients.

## Methods

### Data Collection

Data collected by the EIMS (Epidemiological Investigation of Multiple Sclerosis), (12, 13) GEMS [Genes and Environment in Multiple Sclerosis (13)] and IMSE [Immunomodulation of Multiple Sclerosis Epidemiology (14)] studies were used. A sample of MS patients was individually matched with randomly selected individuals without MS by sex, year of birth and region of residence in Sweden using the Total Population Register. The randomly selected individuals were then sent an invitation to participate and were asked to complete questionnaires and to provide biological samples (including a blood sample). Register data was obtained for all participants. For MS patients, the biological sample was taken when they visited their neurologist, whereas the non-MS cohort were required to visit their local primary health care center to provide their sample once they were recruited into the study. Only the register data and blood samples were used for this study. Matched individuals with the necessary genetic data were included, which resulted in a study population of 3,877 MS patients matched to 4,548 individuals without MS.

Registers include information on inpatient and outpatient diagnoses through the National Patient Register (NPR), and prescription dispensations from the Prescribed Drugs Register (PDR). The PDR only contains information on prescriptions, there is no information on over the counter (OTC) purchases. Additionally, demographic information and data on socioeconomic circumstances are available using the Longitudinal Integration Database for Health Insurance and Labor Market Studies (LISA) maintained by Statistics Sweden. Datasets were merged using the unique personal identification number issued to all Swedish residents at birth or immigration.

### Genotyping

Blood samples were used for DNA extraction and genotyping. Genotypes for 331,536 single nucleotide polymorphisms (SNPs) were determined using a previously described Illumina custom array, the MS Replication Chip (International Multiple Sclerosis Genetics Consortium, 2017) (15). SNPs with <2% minor allele frequency, call-rate <98%, or not in Hardy-Weinberg equilibrium among individuals without MS (*p* < 0.0001) were removed from analysis. After quality control, 94,607 SNPs were included in the dataset. Individuals with > 2% failed genotype calls, with increased heterozygosity (> mean + 2 standard deviations), related individuals (increased identity by descent), or individuals where the recorded sex differed from genotype were removed from analysis. Population outliers identified using the SmartPCA program were removed. A principal component analysis (PCA) was conducted using Eigensoft (16, 17) and six PCA components were used to control for population stratification. HLA alleles for MHC class I and II were imputed by the software HLA^*^IMP:02 (18, 19) for all individuals included using genotypes from the MS Replication Chip, which densely covers the MHC region.

### Outcome

Information from the PDR, which began in July 2005, was used to identify when pain medication had been prescribed. Pain medication can be considered as a proxy for pain with a reasonable degree of sensitivity and specificity (1). For identification of overall pain, an outcome was defined if any anatomical therapeutic chemical (ATC) code for pain relief was recorded in the PDR (see Appendix Table 1 for full list of ATC codes). Neuropathic pain was identified by a recorded ATC code for pregabalin, gabapentin, amitriptyline, capsaicin, or nortriptyline. The register does not include over the counter purchases meaning only medication which was prescribed was used in this study.

Pain medications prescribed prior to the date the MS patient was diagnosed and the same date for matched participants without MS were disregarded.

### Statistical Analysis

Logistic regression, with record of prescribed medication for pain (or specifically neuropathic pain) as the dependent variable was used. Possession of the DQB1^*^03:02 allele was included as a covariate, using firstly a binary yes/no variable referring to whether or not the individual had any alleles characterized as DQB1^*^03:02, and secondly considering number of alleles carried by the individual to ascertain whether zygosity association is present. PCA's were included in the base model to correct for population structure, with the adjusted model additionally including the matching factors (sex, region of residence at the date the MS patient was diagnosed within the matched group, and year of birth), and highest educational attainment (categorized into compulsory school or less, upper secondary, or higher education).

Given that pregabalin and gabapentin are commonly prescribed for the treatment of epilepsy, and amitriptyline and nortriptyline are often used in the treatment of depression, two sensitivity analyses which excluded patients with a diagnosis of epilepsy (*n* = 166) and depression (*n* = 575), respectively, were conducted to ensure results did not significantly alter whether or not these patient groups were included. For the main analysis, these patients were retained because it is possible the treatment was given for a dual use. Only prescribed pain medication was included in the outcome, as it is not possible to identify OTC purchases using the registers.

Analysis was undertaken overall, and separately by cohort characteristic (MS or non-MS). Analysis was also stratified by matching factors to assess the possibility of effect modification.

SAS version 9 and Stata version 13 were used for analysis.

## Results

The distribution of characteristics including the matching factors and highest educational attainment were fairly evenly spread between the two cohorts, with a slightly higher percentage of the non-MS cohort achieving further/higher education relative to the MS cohort (45 and 42%, respectively, Table 1). A statistically significantly larger proportion of the non-MS cohort were homozygous for DQB1^*^03:02 (2% compared with 0.8% of the MS cohort). The proportion within each cohort with one allele was comparable for MS and non-MS, with 23 and 24%, respectively, possessing at least one allele.

**Table 1:** Cohort characteristics.

	**MS, *N* (%)**	**Non-MS, *N* (%)**
Overall	3,877 (100)	4,548 (100)
**Sex**		
Men	939 (24)	1,080 (24)
Women	2,938 (76)	3,468 (76)
**Educational attainment**		
Compulsory school or less	474 (12)	474 (10)
Upper secondary	1,755 (45)	2,015 (44)
Further/higher education	1,645 (42)	2,056 (45)
No educational data available	3 (0)	3 (0)
**Genotype**		
0 DQB1*0302 alleles	2,935 (76)	3,377 (74)
1 DQB1*0302 allele	892 (23)	1,092 (24)
2 DQB1*0302 alleles	32 (1)	79 (2)

Overall, the increased risk of pain medication use for those with one or more DQB1^*^03:02 alleles was not statistically significant, with an adjusted OR of 1.09 (0.98–1.21). After adjustment, the magnitude of the difference between those with and without at least one DQB1^*^03:02 allele was larger for women than for men. The sensitivity analyses which excluded firstly those diagnosed with depression, and subsequently individuals diagnosed with epilepsy, showed no significant differences to the main results.

When considering the results separately for the MS and non-MS cohorts, the presence of the DQB1^*^03:02 genotype appeared to be associated with a greater risk of pain medication use among the non-MS cohort, but not among MS patients. In particular, the presence of the DQB1^*^03:02 genotype increased the strength of the association with pain medication use relative to those without the genotype among non-MS women, with an odds ratio of 1.21 (1.03–1.41). A zygosity effect was evident in the non-MS cohort, with a particularly high magnitude evident for women without MS, jumping from an OR of 1.16 (0.99–1.36) for those heterozygous for the allele relative to those with no DQB1^*^03:02 gene, up to 2.26 (1.25–4.06) for those homozygous for the allele. Such an association was not present for the MS cohort, with no statistically significant associations with pain medication by number of DQB1^*^03:02 alleles (see Tables 3).

For neuropathic pain, the DQB1^*^03:02 genotype was not statistically associated with the risk of pain medication use, although there was a distinct pattern in terms of direction of effect (see Table 2). Differences in zygosity were shown not to be statistically significant.

**Table 2a:** Adjusted only for PCA's.

	**PPM**	**Neuropathic PM**
**Overall**
DQB1^*^0302 –/–	Ref.	
DQB1^*^0302 +/– or +/+	1.07 (0.96–1.18)	1.05 (0.93–1.19)
**Men**
DQB1^*^0302 –/–	Ref.	
DQB1^*^0302 +/– or +/+	1.09 (0.89–1.33)	1.14 (0.88–1.48)
**Women**
DQB1^*^0302 –/–	Ref.	
DQB1^*^0302 +/– or +/+	1.06 (0.94–1.20)	1.04 (0.90–1.20)
**MS**
DQB1^*^0302 –/–	Ref.	
DQB1^*^0302 +/– or +/+	1.02 (0.85–1.23)	1.09 (0.93–1.27)
**MS men**
DQB1^*^0302 –/–	Ref.	
DQB1^*^0302 +/– or +/+	1.10 (0.77–1.56)	1.06 (0.78–1.45)
**MS women**
DQB1^*^0302 –/–	Ref.	
DQB1^*^0302 +/– or +/+	1.00 (0.80–1.24)	1.10 (0.92–1.32)
**Non-MS**
DQB1^*^0302 –/–	Ref.	
DQB1^*^0302 +/– or +/+	1.17 (1.02–1.34)	1.20 (0.94–1.55)
**Non-MS men**
DQB1^*^0302 –/–	Ref.	
DQB1^*^0302 +/– or +/+	1.04 (0.79–1.37)	1.11 (0.58–2.14)
**Non-MS women**
DQB1^*^0302 –/–	Ref.	
DQB1^*^0302 +/– or +/+	1.21 (1.04–1.41)	1.22 (0.93–1.61)

* *Adjusted for PCA's*.

**Table 2b:** Adjusted for PCA's, matching factors, and highest educational attainment.

	**PPM**	**Neuropathic PM**
**Overall**		
DQB1^*^0302 –/–	Ref.	Ref.
DQB1^*^0302 –/+ or +/+	1.09 (0.98–1.21)	1.07 (0.95–1.22)
**Men**		
DQB1^*^0302 –/–	Ref.	Ref.
DQB1^*^0302 –/+ or +/+	1.13 (0.92–1.40)	1.19 (0.90–1.55)
**Women**		
DQB1^*^0302 –/–	Ref.	Ref.
DQB1^*^0302 –/+ or +/+	1.07 (0.95–1.21)	1.04 (0.90–1.20)
**MS**		
DQB1^*^0302 –/–	Ref.	Ref.
DQB1^*^0302 –/+ or +/+	1.02 (0.85-1.23)	1.14 (0.97-1.34)
**MS men**		
DQB1^*^0302 –/–	Ref.	Ref.
DQB1^*^0302 –/+ or +/+	1.06 (0.73–1.54)	1.09 (0.78–1.51)
**MS women**		
DQB1^*^0302 –/–	Ref.	Ref.
DQB1^*^0302 –/+ or +/+	0.99 (0.80–1.24)	1.14 (0.94–1.37)
**Non-MS**		
DQB1^*^0302 –/–	Ref.	Ref.
DQB1^*^0302 –/+ or +/+	1.18 (1.03–1.35)	1.19 (0.93–1.54)
**Non-MS men**		
DQB1^*^0302 –/–	Ref.	Ref.
DQB1^*^0302 –/+ or +/+	1.12 (0.83–1.49)	1.25 (0.63–2.46)
**Non-MS women**		
DQB1^*^0302 –/–	Ref.	Ref.
DQB1^*^0302 –/+ or +/+	1.21 (1.03–1.41)	1.20 (0.91–1.59)

* *Adjusted for PCA's, sex, year of birth, and region of residence, and highest educational attainment*.

**Table 3a:** 2 allele models with PCA adjustment only.

	**PPM**	**Neuropathic PM**
**Overall**
DQB1^*^0302 –/–	Ref.	Ref.
DQB1^*^0302 –/+	1.06 (0.96–1.18)	1.07 (0.94–1.21)
DQB1^*^0302 +/+	1.12 (0.75–1.66)	0.85 (0.52–1.40)
**Men**
DQB1^*^0302 –/–	Ref.	Ref.
DQB1^*^0302 –/+	1.11 (0.90–1.36)	1.17 (0.90–1.52)
DQB1^*^0302 +/+	0.85 (0.43–1.69)	0.70 (0.24–2.01)
**Women**
DQB1^*^0302 –/–	Ref.	Ref.
DQB1^*^0302 –/+	1.05 (0.93–1.19)	1.04 (0.90–1.20)
DQB1^*^0302 +/+	1.30 (0.79–2.13)	0.94 (0.53–1.66)
**MS**
DQB1^*^0302 –/–	Ref.	Ref.
DQB1^*^0302 –/+	1.05 (0.87–1.26)	1.09 (0.93–1.27)
DQB1^*^0302 +/+	0.58 (0.27–1.22)	1.03 (0.49–2.14)
**MS men**
DQB1^*^0302 –/–	Ref.	Ref.
DQB1^*^0302 –/+	1.14 (0.80–1.65)	1.07 (0.78–1.47)
DQB1^*^0302 +/+	0.52 (0.15–1.81)	0.87 (0.23–3.32)
**MS women**
DQB1^*^0302 –/–	Ref.	Ref.
DQB1^*^0302 –/+	1.02 (0.82–1.27)	1.10 (0.92–1.32)
DQB1^*^0302 +/+	0.63 (0.24–1.64)	1.17 (0.48–2.85)
**Non-MS**
DQB1^*^0302 –/–	Ref.	Ref.
DQB1^*^0302 –/+	1.13 (0.99–1.30)	1.18 (0.91–1.53)
DQB1^*^0302 +/+	1.91 (1.20–3.05)	1.55 (0.74–3.26)
**Non-MS men**
DQB1^*^0302 –/–	Ref.	Ref.
DQB1^*^0302 –/+	1.01 (0.76–1.35)	1.11 (0.57–2.19)
DQB1^*^0302 +/+	1.39 (0.62–3.10)	1.10 (0.14–8.52)
**Non-MS women**
DQB1^*^0302 –/–	Ref.	Ref.
DQB1^*^0302 –/+	1.17 (1.00–1.36)	1.19 (0.90–1.58)
DQB1^*^0302 +/+	2.23 (1.25–3.98)	1.71 (0.76–3.84)

* *Adjusted for PCA's*.

**Table 3b:** 2 allele models with PCA adjustment and adjustment for matching factors and highest educational attainment.

	**PPM**	**Neuropathic PM**
**Overall**		
DQB1^*^0302 –/–	Ref.	Ref.
DQB1^*^0302 –/+	1.08 (0.97–1.21)	1.09 (0.95–1.23)
DQB1^*^0302 +/+	1.17 (0.78–1.75)	0.87 (0.53–1.45)
**Men**		
DQB1^*^0302 –/–	Ref.	Ref.
DQB1^*^0302 –/+	1.16 (0.93–1.44)	1.22 (0.93–1.61)
DQB1^*^0302 +/+	0.82 (0.41–1.67)	0.69 (0.24–2.04)
**Women**		
DQB1^*^0302 –/–	Ref.	Ref.
DQB1^*^0302 –/+	1.06 (0.93–1.20)	1.05 (0.90–1.21)
DQB1^*^0302 +/+	1.36 (0.83–2.25)	0.94 (0.53–1.68)
**MS**		
DQB1^*^0302 –/–	Ref.	Ref.
DQB1^*^0302 –/+	1.05 (0.86–1.27)	1.14 (0.97–1.34)
DQB1^*^0302 +/+	0.60 (0.28–1.31)	1.18 (0.56–2.50)
**MS men**		
DQB1^*^0302 –/–	Ref.	Ref.
DQB1^*^0302 –/+	1.12 (0.76–1.64)	1.10 (0.78–1.54)
DQB1^*^0302 +/+	0.44 (0.12–1.59)	0.88 (0.22–3.52)
**MS women**		
DQB1^*^0302 –/–	Ref.	Ref.
DQB1^*^0302 –/+	1.01 (0.81–1.26)	1.13 (0.94–1.36)
DQB1^*^0302 +/+	0.67 (0.25–1.79)	1.34 (0.54–3.31)
**Non-MS**		
DQB1^*^0302 –/–	Ref.	Ref.
DQB1^*^0302 –/+	1.14 (0.99–1.31)	1.17 (0.90–1.53)
DQB1^*^0302 +/+	1.97 (1.22–3.18)	1.49 (0.69–3.22)
**Non-MS men**		
DQB1^*^0302 –/–	Ref.	Ref.
DQB1^*^0302 –/+	1.09 (0.81–1.48)	1.23 (0.61–2.48)
DQB1^*^0302 +/+	1.41 (0.59–3.39)	1.39 (0.17–11.41)
**Non-MS women**		
DQB1^*^0302 –/–	Ref.	Ref.
DQB1^*^0302 –/+	1.16 (0.99–1.36)	1.17 (0.88–1.56)
DQB1^*^0302 +/+	2.26 (1.25–4.06)	1.62 (0.70–3.71)

* *Adjusted for PCA's and matching factors and highest educational attainment*.

## Discussion

The expected increased risk of pain for individuals possessing the DQB1^*^03:02 allele was observed for those without MS, but not among MS patients. For neuropathic pain, the findings were null, with no association between the DQB1^*^03:02 genotype and pain for either the MS or non-MS group.

One possibility for why there was no association between the DQB1^*^03:02 genotype and pain among MS patients could relate to the fact that levels of pain are high in MS patients regardless of genotype (1), resulting in a smaller magnitude effect of genotype. Although this is the case for MS patients, who already experience heightened inflammation of the CNS, the same is not found for individuals without MS, perhaps indicating a lower base level of inflammation and pain could result in a stronger effect of genotype. The HLA-DQB1 genes have previously been associated with autoimmune disorders and other inflammatory processes (20), perhaps giving insight into why pain may be increased among individuals without MS with the genotype DQB1^*^03:02 (21). Previously, inflammatory processes have been linked to pain, especially neuropathic pain (22). Pro-inflammatory cytokine levels have been shown to be associated with the likelihood of pain treatment being successful (23), with individuals with low grade inflammation more likely to report improvements to pain levels when undergoing behavioral treatment.

The possibility that higher levels of inflammatory markers increases the risk of pain could also have implications for sex differences in pain, with previous studies documenting women tend to report higher levels of pain, and have higher levels of inflammation, including in the form of autoimmune diseases (24). Previous studies into pain and inflammation indicate that the effect of a particular exposure on pain could be mediated through the effect of inflammation, particularly CNS inflammation when considering MS patients (9). If the mechanism by which DQB1^*^03:02 acts on pain is through inflammation, it suggests a mediating effect is in action rather than the gene acting directly on the propensity for pain within the non-MS group. However, the effect of the gene on immune and inflammatory responses are complex. With respects to the heterodimer produced by DQA1 and DQB1 genes, changes to the dimer as a result of SNPs in the DQA1 and DQB1 alleles, such as DQB1^*^03:02, could affect the binding affinity of the dimer for antigen presentation (10). Different dimers could contribute differentially to inflammatory processes, and to pain. Further research is needed in order to disentangle whether this is a possibility.

When considering a possible association between risk of pain and DQB1^*^03:02 zygosity, the findings highlighted that such an association was present for the non-MS cohort, driven primarily by a stronger association with zygosity in women, but not for the MS cohort. Again this could relate to increased risk of pain medication use for MS patients regardless of the number of DQB1^*^03:02 alleles, reducing the strength of the association between genotype and pain. Proportionally more non-MS individuals possessed two DQB1^*^03:02 alleles relative to those with MS, although percentages with the two allele genotype were relatively rare (2% of the non-MS cohort and 0.8% of the MS cohort). In addition, MS pain likely stems from lesions in the CNS, and our data suggest that there is no major genetic influence on this MS sub-phenotype, and may mainly be dependent on stochastic factors determining the location of MS lesions.

An important finding of the study is the apparent differential effect of the presence of the DQB1^*^03:02 genotype on pain medication use for women relative to men amongst the non-MS cohort, suggesting sex acts as an effect modifier. It has been previously documented that women are more likely to experience or report pain than men (25, 26), a finding replicated in this study, although the mechanisms behind this are incompletely understood. One possible explanation for at least increased reporting of pain amongst women could relate to the higher levels of healthcare utilization seen among women relative to men (27), which would also most likely result in higher levels of pain relief prescriptions. Such a process could result in more records of prescriptions even if pain levels are similar among men and women with the DQB1^*^03:02 genotype. Whilst the effect of the DQB1^*^03:02 genotype on the association with pain appears to be stronger for women than men, the smaller number of observations amongst men may mean this finding is partially driven by a lack of power amongst men, so results should be interpreted with caution.

Alongside considering pain in a more general sense, this study also investigated specifically neuropathic pain in relation to the presence or absence of the DQB1^*^03:02 allele. Overall, similar patterns were identified for neuropathic pain in terms of the effect of the DQB1^*^03:02 allele for the non-MS group, however fewer events (since those identified as having neuropathic pain were a subgroup of those identified as having pain overall) meant reduced power to detect associations, which may be why results for this pain phenotype were not statistically significant.

Interestingly, the findings of this study mirrors several features previously described in experimental models of pain. The susceptibility to develop neuropathic pain-like behavior among inbred and congenic rat strains subjected to a standardized peripheral nerve injury has been shown to be linked to the MHC (6, 7). This effect, however, was not discernible with injuries to the spinal cord, which is more similar to the central nervous system involvement seen in MS (8). Furthermore, female rats displayed increased susceptibility to neuropathic pain-like behavior after a standardized injury to the spinal cord (28), which mapped to several parts of the genome and some of which displayed sex-specific regulation. Collectively, these observations provide support for a different genetic regulation of pain occurring after injuries to the peripheral and central nervous systems, respectively, and that some of those genetic influences are sex regulated, akin to what was observed here.

This study had several strengths. The definition of exposure to MS was robust and required attendance at an MS clinic, improving specificity. The ability to identify pain through prescribed pain medications allowed an objective approach to measurement of the outcome rather than, for example, questionnaires being used (29). The use of Swedish registers meant there was no loss to follow-up. Registers, including the NPR and PDR are regularly updated and require no response directly from participants.

Some potential limitations to the study should also be considered. MS patients were recruited during their visits to the neurologist, when blood samples used for genetic profiling were collected. Non-MS study recruits had to attend their local health center to deposit their blood samples, which may result in bias to the health profiles of the non-MS group. Individuals with frequent visits to health centers due to health problems may be more likely to participate, due to frequent contact with health services. Conversely, those with mobility problems or poorer health may be less inclined to visit the health center to provide their sample, so bias could be acting in both directions, making it difficult to estimate how results might be affected. The sampling of MS patients is not based on probability sampling, but is rather convenience sampling whereby patients agree to participate and are then recruited into the study during their visits with an MS specialist. This could result in selection bias, and impact on the generalizability of the results, because self-selecting samples are not reflective of the population (30, 31). In addition only using prescribed pain medications to define the outcome is potentially limiting, and likely leads to an underestimate of the true number of individuals experiencing pain (32) and it may also tend to identify more severe and persistent pain, as this is more likely to result in receipt of prescribed medication. Finally, gabapentin can be prescribed for spasticity rather than specifically for pain. This could therefore reduce the specificity of the outcome. However, gabapentin is not often used as a monotherapy (33), and is generally prescribed when patients present with symptoms suggestive of neuropathic pain, along with spasticity (34).

Overall, our results indicated no association between possessing the DQB1^*^03:02 genotype and pain in patients with MS, however, the risk of pain medication was increased, albeit modestly, for those with one or more DQB1^*^03:02 alleles among the non-MS cohort. Furthermore, this finding was strengthened by an association with zygosity among the non-MS cohort, particularly for women. The same pattern was seen with neuropathic pain, however smaller numbers reduced power and makes conclusions difficult due to a lack of significance. Although the HLA complex also includes non-immune genes, a growing body of evidence suggest that immune functions are involved in the development of pain and the findings presented here further strengthen this notion.

## Data Availability Statement

The data that support the findings of this study are available from the Swedish National Board of Health and Welfare (Prescribed Drug Register, National Patient Register, and Swedish Medical Birth Register), Karolinska Institute (MS register), and Statistics Sweden (Total Population Register). The register data supporting these findings can be obtained by applying to the relevant register holders as listed above. The data for EIMS, GEMS, and IMSE can be obtained by applying to the data holders. Enquiries can be sent to Jan Hillert (jan.hillert@ki.se).

## Ethics Statement

The studies involving human participants were reviewed and approved by Regional ethics committee of Stockholm. The patients/participants provided their written informed consent to participate in this study.

## Author Contributions

SBu conducted the data analysis and wrote the initial draft of the manuscript. KS and PS worked with SBu on the drafting of the first draft, and were involved in interpretation of the data and provided scientific input. IK was involved in the data application, interpretation of results and provided comments on the manuscript. JH was involved in the data application, interpretation of results and provided comments on the manuscript. HL was involved in the interpretation of results, and provided comments on the manuscript. LA was involved in the data application, interpretation of results and provided comments on the manuscript. TO was involved in the data application, interpretation of results and provided comments on the manuscript. FP was involved in the data application, interpretation of results and provided comments on the manuscript. SM was involved in the data application, interpretation of results and provided comments on the manuscript. SBa was involved in the data application, interpretation of results and provided comments on the manuscript. All authors contributed to the article and approved the submitted version.

## Conflict of Interest

FP has received unrestricted academic research grants from Biogen, Genzyme, Novartis, and received research grants from Genzyme, Merck KGaA, and Novartis, and fees for serving as Chair of DMC in clinical trials with Parexel. IK has received funding from AstraZeneca, and a speaker honorary from MercSernono. JH has received funding from Biogen, Novartis, Merck-Serono, Bayer-Schering, and Teva and has received speaker honoraria from Sanofi-Aventis. LA has received speaker honoraria from Teva. TO has received funding from AstraZeneca, and speaker and advisory board honoraria from Biogen, Novartis, and Genzyme. SM has received funding from AstraZeneca, and funding for MS related research from F. Hoffman-La Roche AG, and Novartis International AG. The remaining authors declare that the research was conducted in the absence of any commercial or financial relationships that could be construed as a potential conflict of interest.

## Appendix 1

**Table:** ATC codes used to identify pain type.

**ATC codes**	
All pain medication	N02A N02BE M03BB M03BC N02BA N03AX12 N03AX16 N02C N02BG10 N06AA09 N06AA10
Neuropathic	N03AX12 N03AX16 M02AB N06AA09 N06AA10

## References

[1] Burkill S Montgomery S Kockum I Piehl F Strid P Hillert J. The association between multiple sclerosis and pain medications. Pain. (2019) 160:424–32. 10.1097/j.pain.0000000000001429 30376533

[2] Khan N Smith MT. Multiple sclerosis-induced neuropathic pain: pharmacological management and pathophysiological insights from rodent EAE models. Inflammopharmacology. (2014) 22:1–22. 10.1007/s10787-013-0195-3 24234347 PMC3933737

[3] Moutsianas L Jostins L Beecham AH Dilthey AT Xifara DK Ban M. Class II HLA interactions modulate genetic risk for multiple sclerosis. Nat Genet. (2015) 47:1107–13. 10.1038/ng.3395 26343388 PMC4874245

[4] International Multiple Sclerosis Genetics C Beecham AH Patsopoulos NA Xifara DK Davis MF Kemppinen A. Analysis of immune-related loci identifies 48 new susceptibility variants for multiple sclerosis. Nat Genet. (2013) 45:1353–60. 10.1038/ng.2770 24076602 PMC3832895

[5] International Multiple Sclerosis Genetics C, Wellcome Trust Case Control C Sawcer S Hellenthal G Pirinen M Spencer CC. Genetic risk and a primary role for cell-mediated immune mechanisms in multiple sclerosis. Nature. (2011) 476:214–9. 10.1038/nature10251 21833088 PMC3182531

[6] Dominguez CA Li L Lidman O Olsson T Wiesenfeld-Hallin Z Piehl F. Both MHC and non-MHC genes regulate development of experimental neuropathic pain in rats. Neurosci Lett. (2008) 442:284–6. 10.1016/j.neulet.2008.07.027 18640240

[7] Dominguez CA Lidman O Hao JX Diez M Tuncel J Olsson T. Genetic analysis of neuropathic pain-like behavior following peripheral nerve injury suggests a role of the major histocompatibility complex in development of allodynia. Pain. (2008) 136:313–9. 10.1016/j.pain.2007.07.009 17764842

[8] Dominguez CA Lidman O Olsson T Wiesenfeld-Hallin Z Piehl F Xu XJ. Contrasting genetic effects of major histocompatibility complex on ischemic peripheral nerve and spinal cord injury in female rats. Neurosci Lett. (2008) 443:95–8. 10.1016/j.neulet.2008.07.063 18675884

[9] Ji RR Chamessian A Zhang YQ. Pain regulation by non-neuronal cells and inflammation. Science. (2016) 354:572–7. 10.1126/science.aaf8924 27811267 PMC5488328

[10] Kwok WW Mickelson E Masewicz S Milner EC Hansen J Nepom GT. Polymorphic DQ alpha and DQ beta interactions dictate HLA class II determinants of allo-recognition. J Exp Med. (1990) 171:85–95. 10.1084/jem.171.1.85 2104922 PMC2187655

[11] Muller AE Clausen T Sjogren P Odsbu I Skurtveit S. Prescribed opioid analgesic use developments in three Nordic countries, 2006–2017. Scand J Pain. (2019) 19:345–53. 10.1515/sjpain-2018-0307 30677000

[12] Persson A Epidemiological Investigation of Multiple Sclerosis – EIMS. (2016). Available online at: http://ki.se/en/imm/epidemiological-investigation-of-multiple-sclerosis-eims (accessed May 29, 2017).

[13] Hedstrom AK Hillert J Olsson T Alfredsson L. Alcohol as a modifiable lifestyle factor affecting multiple sclerosis risk. Jama Neurol. (2014) 71:300–5. 10.1001/jamaneurol.2013.5858 24395432

[14] Frisell T Forsberg L Nordin N Kiesel C Alfredsson L Askling J. Comparative analysis of first-year fingolimod and natalizumab drug discontinuation among Swedish patients with multiple sclerosis. Mult Scler. (2016) 22:85–93. 10.1177/1352458515579216 25921036

[15] International Multiple Sclerosis Genetics Consorti NP Baranzini SE Patsopoulos NA Santaniello A Shoostari P Cotsapas C. The multiple sclerosis genomic map: role of peripheral immune cells and resident 1 microglia in susceptibility. bioRxiv. (2017). Available online at: https://www.biorxiv.org/content/biorxiv/early/2017/07/13/143933.full.pdf

[16] Price AL Patterson NJ Plenge RM Weinblatt ME Shadick NA Reich D. Principal components analysis corrects for stratification in genome-wide association studies. Nat Genet. (2006) 38:904–9. 10.1038/ng1847 16862161

[17] Patterson N Price AL Reich D. Population structure and eigenanalysis. PLoS Genet. (2006) 2:e190. 10.1371/journal.pgen.0020190 17194218 PMC1713260

[18] Dilthey AT Moutsianas L Leslie S McVean G. HLA^*^IMP-an integrated framework for imputing classical HLA alleles from SNP genotypes. Bioinformatics. (2011) 27:968–72. 10.1093/bioinformatics/btr061 21300701 PMC3065693

[19] Dilthey A Leslie S Moutsianas L Shen JD Cox C Nelson MR. Multi-population classical HLA type imputation. Plos Comput Biol. (2013) 9:e1002877. 10.1371/journal.pcbi.1002877 23459081 PMC3572961

[20] Lincoln MR Ramagopalan SV Chao MJ Herrera BM DeLuca GC Orton SM. Epistasis among HLA-DRB1, HLA-DQA1, and HLA-DQB1 loci determines multiple sclerosis susceptibility. Procee Natl Acad Sci USA. (2009) 106:7542–7. 10.1073/pnas.0812664106 19380721 PMC2678609

[21] Mangalam A Luckey D Basal E Jackson M Smart M Rodriguez M. HLA-DQ8 (DQB1^*^0302)-restricted Th17 cells exacerbate experimental autoimmune encephalomyelitis in HLA-DR3-transgenic mice. J Immunol. (2009) 182:5131–9. 10.4049/jimmunol.0803918 19342694 PMC2665933

[22] Sommer C Leinders M Uceyler N. Inflammation in the pathophysiology of neuropathic pain. Pain. (2018) 159:595–602. 10.1097/j.pain.0000000000001122 29447138

[23] Lasselin J Kemani MK Kanstrup M Olsson GL Axelsson J Andreasson A. Low-grade inflammation may moderate the effect of behavioral treatment for chronic pain in adults. J Behav Med. (2016) 39:916–24. 10.1007/s10865-016-9769-z 27469518 PMC5012257

[24] Derry HM Padin AC Kuo JL Hughes S Kiecolt-Glaser JK. Sex differences in depression: does inflammation play a role? Curr Psychiatry Rep. (2015) 17:78. 10.1007/s11920-015-0618-5 26272539 PMC4869519

[25] Wiesenfeld-Hallin Z. Sex differences in pain perception. Gender Med. (2005) 2:137–45. 10.1016/S1550-8579(05)80042-7 16290886

[26] Younger JW. Being female is a risk factor for chronic pain. Are inflammatory processes to blame? Brain Behav Immun. (2015) 46:33–4. 10.1016/j.bbi.2015.01.006 25596175

[27] Redondo-Sendino A Guallar-Castillon P Banegas JR Rodriguez-Artalejo F. Gender differences in the utilization of health-care services among the older adult population of Spain. BMC Public Health. (2006) 6:155. 10.1186/1471-2458-6-155 16780576 PMC1525176

[28] Dominguez CA Strom M Gao T Zhang L Olsson T Wiesenfeld-Hallin Z. Genetic and sex influence on neuropathic pain-like behaviour after spinal cord injury in the rat. Eur J Pain. (2012) 16:1368–77. 10.1002/j.1532-2149.2012.00144.x 22473909

[29] Althubaiti A. Information bias in health research: definition, pitfalls, and adjustment methods. J Multidiscip Healthc. (2016) 9:211–7. 10.2147/JMDH.S104807 27217764 PMC4862344

[30] Markovic G Schult ML Bartfai A. The effect of sampling bias on generalizability in intervention trials after brain injury. Brain Inj. (2017) 31:9–15. 10.1080/02699052.2016.1206213 27819515

[31] Erens B Burkill S Couper MP Conrad F Clifton S Tanton C. Nonprobability web surveys to measure sexual behaviors and attitudes in the general population: a comparison with a probability sample interview survey. J Med Internet Res. (2014) 16:e276. 10.2196/jmir.3382 25488851 PMC4275497

[32] Ehde DM Alschuler KN Osborne TL Hanley MA Jensen MP Kraft GH. Utilization and patients' perceptions of the effectiveness of pain treatments in multiple sclerosis: a cross-sectional survey. Disabil Health J. (2015) 8:452–6. 10.1016/j.dhjo.2015.03.001 25899795 PMC4464976

[33] Chang E Ghosh N Yanni D Lee S Alexandru D Mozaffar T. A review of spasticity treatments: pharmacological and interventional approaches. Crit Rev Phys Rehabil Med. (2013) 25:11–22. 10.1615/CritRevPhysRehabilMed.2013007945 25750484 PMC4349402

[34] Keenan E Spasticity management, part 2: Choosing the right medication to suit the individual. Br J Neurosci Nurs. (2009) 5:419–24. 10.12968/bjnn.2009.5.9.44099

